# NSC-87877 inhibits DUSP26 function in neuroblastoma resulting in p53-mediated apoptosis

**DOI:** 10.1038/cddis.2015.207

**Published:** 2015-08-06

**Authors:** Y Shi, I T Ma, R H Patel, X Shang, Z Chen, Y Zhao, J Cheng, Y Fan, Y Rojas, E Barbieri, Z Chen, Y Yu, J Jin, E S Kim, J M Shohet, S A Vasudevan, J Yang

**Affiliations:** 1 Division of Pediatric Surgery, Texas Children's Hospital Department of Surgery, Michael E. DeBakey Department of Surgery, Dan L. Duncan Cancer Center, Baylor College of Medicine, Clinical Care Center, Houston, TX, USA; 2 Department of Surgery, Mayo Clinic Arizona, Phoenix, AZ, USA; 3 Texas Children's Cancer Center, Department of Pediatrics, Dan L. Duncan Cancer Center, Baylor College of Medicine, Houston, TX, USA; 4 Division of Pediatric Surgery, Department of Surgery, Keck School of Medicine, University of Southern California, Los Angeles, CA, USA

## Abstract

Dual specificity protein phosphatase 26 (DUSP26) is overexpressed in high-risk neuroblastoma (NB) and contributes to chemoresistance by inhibiting p53 function. *In vitro*, DUSP26 has also been shown to effectively inhibit p38 MAP kinase. We hypothesize that inhibiting DUSP26 will result in decreased NB cell growth in a p53 and/or p38-mediated manner. NSC-87877 (8-hydroxy-7-[(6-sulfo-2-naphthyl)azo]-5-quinolinesulfonic acid), a novel DUSP26 small molecule inhibitor, shows effective growth inhibition and induction of apoptosis in NB cell lines. NB cell lines treated with small hairpin RNA (shRNA) targeting DUSP26 also exhibit a proliferation defect both *in vitro* and *in vivo*. Treatment of NB cell lines with NSC-87877 results in increased p53 phosphorylation (Ser37 and Ser46) and activation, increased activation of downstream p38 effector proteins (heat shock protein 27 (HSP27) and MAP kinase-activated protein kinase 2 (MAPKAPK2)) and poly ADP ribose polymerase/caspase-3 cleavage. The cytotoxicity resulting from DUSP26 inhibition is partially reversed by knocking down p53 expression with shRNA and also by inhibiting p38 activity with SB203580 (4-[4-(4-fluorophenyl)-2-(4-methylsulfinylphenyl)-1*H*-imidazol-5-yl]pyridine). In an intrarenal mouse model of NB, NSC-87877 treatment results in decreased tumor growth and increased p53 and p38 activity. Together, these results suggest that DUSP26 inhibition with NSC-87877 is an effective strategy to induce NB cell cytotoxicity *in vitro* and *in vivo* through activation of the p53 and p38 mitogen-activated protein kinase (MAPK) tumor-suppressor pathways.

Neuroblastoma (NB) remains the most common extracranial solid tumor in children and is associated with a very poor prognosis in high-risk patients.^1^ Current treatment strategy for this subgroup of patients includes intense myeloablative chemotherapy, radical surgical resection of primary tumor, radiation therapy and stem cell rescue. In spite of these therapies, survival in high-risk NB patients is <50% at 5 years from diagnosis.^2^ These therapies confer major long-term toxicities in over 90% of long-term survivors; therefore, efforts are underway to identify more specific biologic therapies with less toxicity and better efficacy at targeting NB.

A therapeutic strategy that is gaining much interest is utilizing small molecule inhibitors to activate innate, non-mutated cell senescence and death pathways, such as the p53 tumor suppressor. Mutations in the p53 gene are seen in over 60% of adult cancers; however, pediatric solid tumors, particularly NB, do not exhibit frequent p53 mutations and actually have an intact pathway that is suppressed by other mechanisms.^3^ Mouse double minute 2 (MDM2) inhibition is a strategy to activate p53 using compounds such as Nutlin-3a, RITA and RG7112, which has already been tested in a phase I clinical trial in adults.^4, 5, 6^ The p38 stress kinase, MAP kinase, pathway is another tumor-suppressive pathway that is upstream from p53 and can function through p53-dependent and -independent mechanisms to induce apoptosis. Although described as oncogenic in some cancers, there is evidence that p38 activation leads to tumor cell apoptosis in NB.^7, 8, 9, 10^ Both of these tumor-suppressive pathways are regulated through phosphorylation and dephosphorylation events by an array of kinases and phosphatases.

Phosphatase targeting in NB has had very limited application because of the limited number of phosphatases found to have an oncogenic role. Protein phosphatase 2A (PP2A), protein tyrosine phosphatase receptor delta (PTPRD) and dual specificity protein phosphatase 12 (DUSP12) have been found to be involved in NB cell differentiation and tumor suppression.^11, 12, 13, 14^ First discovered in breast cancer, PPM1D, or Wip-1 phosphatase, is active in NB, and small molecule inhibition results in p53 activation and chemosensitivity.^15, 16, 17^ In this report, we show DUSP26 functions by inhibiting p53 and p38 function to promote growth of NB tumor cells.

DUSP26 (MKP-8, LDP-4) was originally described as a dual specifity phosphatase with enzymatic activity against p38 MAP kinase resulting in dephosphorlyation of the primary p38 activation sites, Thr180/Tyr182.^18, 19^ Song *et al.*^20^ showed that NSC-87877 (8-hydroxy-7-[(6-sulfo-2-naphthyl)azo]-5-quinolinesulfonic acid), a small molecule phosphatase inhibitor of SHP-1 (src homology region 2 domain-containing phosphatase), specifically inhibits DUSP26 phosphatase activity at a much lower IC_50_ than other phosphatases resulting in increased p38 phosphorylation *in vitro*. Yu *et al.*^21^ have shown that DUSP26 is overexpressed in anaplastic thyroid cancer tissue samples and functions by inhibiting the p38 MAP kinase pathway.

A novel DUSP26 function shown in NB is enzymatic regulation of the p53 tumor suppressor.^22^ We demonstrated that DUSP26 physically binds p53, dephosphorylates p53 at Ser20 and Ser37, and causes inhibition of downstream p53 signaling. DUSP26 activity leads to increased chemoresistance to doxorubicin and VP-16 (etoposide) treatment, and overexpression was seen in high-risk NB tumor tissue samples correlating with a worse prognosis in these patients. Here, we show how DUSP26 has pro-proliferative effects in NB by inhibiting the p53 tumor-suppressor pathway, as well as the p38 mitogen-activated protein kinase (MAPK) pathway. Inhibition with small hairpin RNA (shRNA) targeting DUSP26 or NSC-87877 results in decreased proliferation and cell viability in NB cell lines *in vitro* and *in vivo*. Inhibiting the p53 or p38 pathways reverses this phenotype seen with inhibition of DUSP26. These data establish DUSP26 inhibition as a promising novel therapeutic approach for NB.

## Results

### NSC-87877 inhibits DUSP26 function in NB cell lines resulting in decreased proliferation

**Figure 1:**
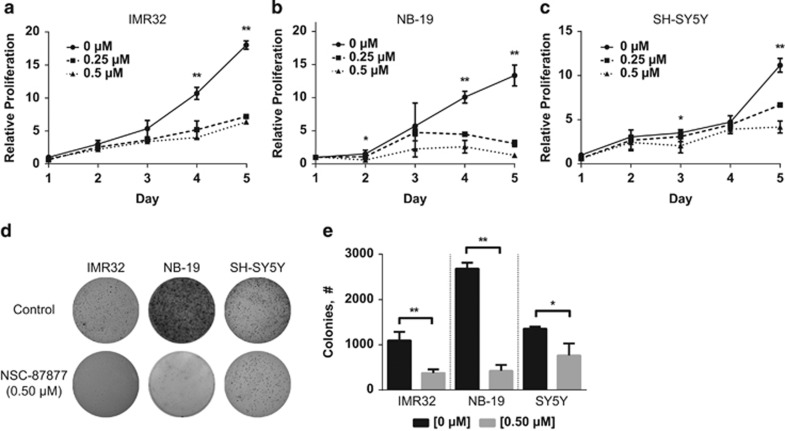
NSC-87877 shows decreased cell proliferation in NB cell lines. (**a**–**c**) Three NB cell lines, IMR32, NB-19 and SH-SY5Y, were treated with NSC-87877 at the indicated concentrations. Cell proliferation was performed using MTT and measuring absorbance at 24 h, and at every subsequent 24 h for 5 days. Data points were compared as a fold change relative to day 1. Data were represented as a mean±S.D. *P*-values <0.05 for (0) *versus* (0.25 and 0.5) (**), <0.05 for (0) *versus* (0.5) (*). (**d**) Three NB cell lines, IMR32, NB-19 and SH-SY5Y, were seeded in six-well plates with NSC-87877, media and agar, and then grown for 2 weeks. The colonies were stained with MTT for 4 h and pictures were taken. (**e**) Colonies were counted and shown as mean±S.D. *P*-values <0.05 (*) or <0.01 (**) are indicated. Panels **a** to **c** are representative of three independent experiments. Panels **d** and **e** are representative of two independent experiments

To test the effects of NSC-87877, a chemical inhibitor of DUSP26, we exposed three p53 wild-type NB cell lines, IMR32, NB-19 and SH-SY5Y, known to express DUSP26 (Supplementary Figure 1) with low concentrations of the chemical inhibitor. In all cell lines, we saw a decrease in cell proliferation by day 5 when exposed to 0.25 *μ*M of NSC-87877 (Figures 1a–c). In NB-19 and SH-SY5Y, the decrease in cell proliferation seemed more pronounced with a 0.5 *μ*M dose. The same cell lines were then plated in soft agar, which recapitulates *in vivo* tumor growth to a greater degree than two-dimensional cell growth,^23^ with 0.5 *μ*M of NSC-87877 and grown for 14 days. After staining with MTT (3-(4,5-dimethylthiazol-2-yl)-2,5-diphenyltetrazolium bromide), the colony formation was significantly decreased in the three cell lines tested when compared with the control group (Figures 1d–e, *P*<0.01). In order to demonstrate that NSC-87877 can inhibit DUSP26 function in NB cell lines, we used the SK-N-AS cell line that expresses DUSP26 and transduced this cell line with a DUSP26 overexpression construct (Supplementary Figure 2a). DUSP26 overexpression in SK-N-AS causes a decrease in p38 MAPK phosphorylation as previously demonstrated in HEK293T cells.^18, 20^ However, when this cell line was treated with NSC-87877, we saw an increase in p38 phosphorylation indicating that DUSP26 function is inhibited. This finding is consistent with the results reported by Song *et al.^20^* describing NSC-87877 as a DUSP26 inhibitor.

### DUSP26 shRNA-treated NB cell lines also display a proliferation defect

**Figure 2:**
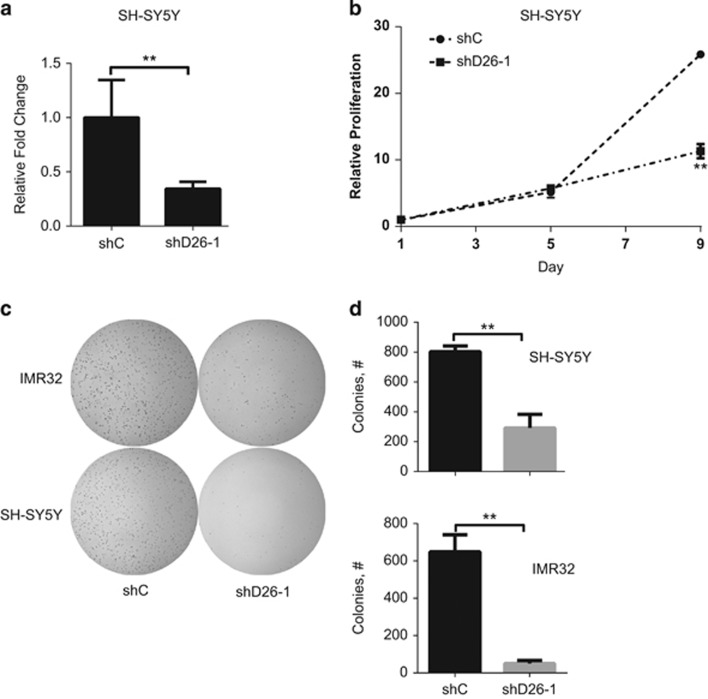
Knockdown of DUSP26 causes decreased cell proliferation in NB cell lines. (**a**) SH-SY5Y was transduced using an shRNA construct targeting DUSP26 (shD26-1) as well as a non-silencing construct. Real-time RT-PCR was used to quantify DUSP26 mRNA. Results were presented as relative fold change compared with shC±S.D. *P*-value of <0.01 (**) is indicated. (**b**) SH-SY5Y transduced with shC and shD26-1 were each seeded into individual 96-well plates. Cell proliferation was measured with CCK-8, absorbance was read at 24 h, and at every subsequent 24 h for 9 days. Data points were compared as a fold change relative to day 1. Results are presented as mean±S.D. *P*-value <0.01 (**) is indicated. (**c**) SH-SY5Y and IMR32 transduced with shC and shD26-1 were each seeded into individual six-well plates to measure anchorage independent growth using a soft agar assay, then incubated for 3 weeks. The colonies were stained with MTT for 4 h and pictures were taken. (**d**) Colonies were counted and shown as mean±S.D. *P*-values <0.01 (**) are indicated. All data in this figure are representative of three independent experiments

In order to validate the above data, we designed shRNA targeting DUSP26 to achieve decreased mRNA expression. Figure 2a shows a >50% expression knockdown with the shDUSP26-1 (shD26-1) sequence in the SH-SY5Y cell line. A non-targeting, control shRNA (shC) sequence was used as a comparison. These cell lines were then grown and tested for proliferation. At day 9, there was a significant difference comparing the shC cell line to shD26-1 (*P*<0.01). This was also true in the IMR32 cell line tested in a similar manner (Supplementary Figures 3a and b). Two additional DUSP26 shRNA's were designed and found to have significant inhibition of DUSP26 expression and both caused decreased proliferation (Supplementary Figures 3c and d). We then tested the effect of DUSP26 knockdown in a soft agar assay. Similar to what was seen with NSC-87877 treatment in soft agar, the shD26-1 cell line showed significantly less colonies (*P*<0.01) than the shC cell line in both SH-SY5Y and IMR32 (Figures 2c and d).

As NSC-87877 was originally described as an inhibitor of Src homology region 2 domain-containing phosphatases (SHP-1 and SHP-2), we tested the effects of shRNA targeting SHP-1 in SH-SY5Y. Using a previously published shRNA sequence targeting SHP-1 (shSHP-1),^24^ gene expression was decreased per immunoblot (Supplementary Figure 4a). These cells did not display decreased proliferation when compared with control cells and shD26-1 (Supplementary Figure 4b); however, when grown in soft agar, the shSHP-1 cells made much larger colonies than the control further indicating a lack of a proliferation defect and very different phenotype than DUSP26 inhibition (Supplementary Figure 4c).

### DUSP26 shRNA causes decreased tumor growth *in vivo*

**Figure 3:**
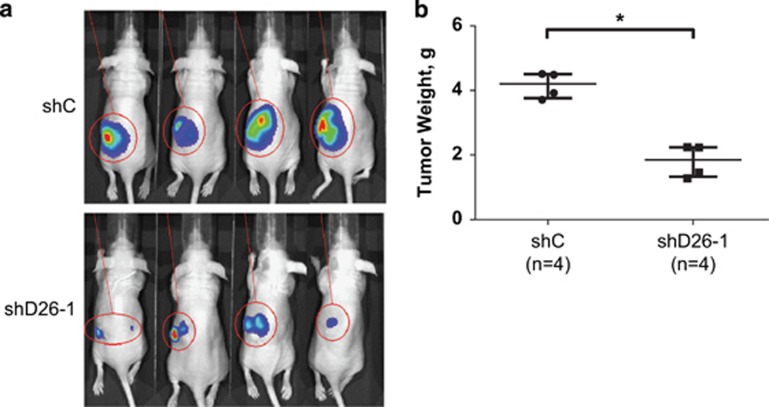
Knockdown of DUSP26 inhibits NB growth *in vivo*. (**a**) SH-SY5Y expressing luciferase were transduced with shC or shD26-1 and implanted into the left kidney of female NCr nude mice. Bioluminescent images were taken every week after i.p. injection of luciferin. (**b**) After 4 weeks, necropsy was performed, and tumors were extracted and weighed. The data are presented as median±one quartile, and individual data points are shown. *P*-values <0.05 (*) are indicated

As the soft agar colony formation assay mimics *in vivo* tumor growth, we tested the shD26-1 *versus* the shC sequence in an intrarenal model of NB using the SH-SY5Y cell line with luciferase expression. After transduction and selection of these cell lines, female nude mice were injected with 1 × 10^6^ cells into the left kidney and allowed to grow. Tumor growth was monitored periodically with intraperitoneal (i.p.) injections of luciferin and bioluminescence images were taken showing a decrease in tumor size of the shD26-1 cell line compared with shC (Figure 3a). At 4 weeks, a necropsy was performed and the tumors were weighed. The shD26-1 tumors weighed significantly less than the shC tumors (*P*<0.05; Figure 3b).

### NSC-87877 can induce apoptosis in NB cell lines

**Figure 4:**
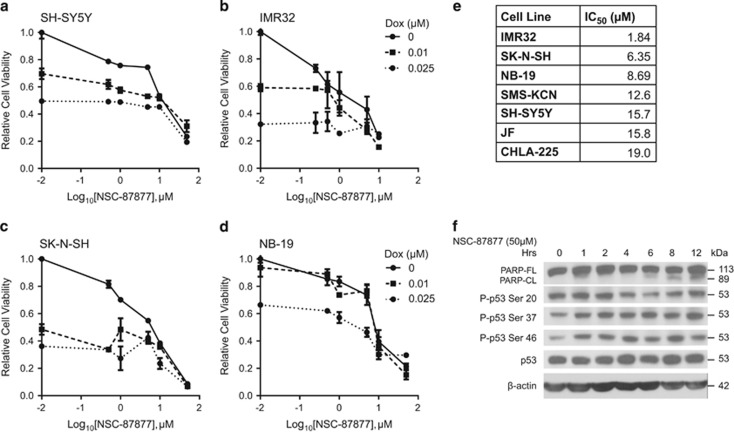
NSC-87877 induces apoptosis in NB cell lines *in vitro*. (**a-d**) A panel of four NB cell lines, SH-SY5Y, IMR32, SK-N-SH and NB-19 were seeded into 96-well plates. Each cell line was treated with the indicated concentrations of doxorubicin (Dox) and NSC-87877. After 24–48 h, cells were stained with MTT for 4 h and absorbance was read at 540 nm. The data are presented as mean±S.D. (**e**) A panel of seven NB cell lines were seeded into 96-well plates and treated with NSC-87877 at the indicated concentrations. After 24–48 h, cell viability was measured by staining with MTT for 4 h and reading the absorbance at 540 nm. The IC_50_ of NSC-87877 was calculated using nonlinear regression in Prism (Graphpad, San Diego, CA, USA). (**f**) IMR32 cells were treated with 50 *μ*M NSC-87877 for the indicated durations. Immunoblotting was performed on using PARP, phospho-p53 serine 20, phospho-p53 serine 37, phosphor-p53 serine 46 and p53 antibodies. *β*-Actin was used as a loading control. Panels **a**–**e** are representative of three independent experiments. Panel **f** is representative of two independent experiments

Having shown that nanomolar doses of NSC-87877 can result in a proliferation defect in NB cell lines similar to shRNA knockdown of DUSP26, we tested higher doses of NSC-87877 on NB to see if cytotoxicity could be induced. As seen in our previous studies using shRNA targeting DUSP26,^22^ concomitant treatment with NSC-87877 and doxorubicin resulted in enhanced cytotoxicity when compared with doxorubicin treatment alone (Figures 4a–d). As a single agent, we found that increasing doses of NSC-87877 resulted in apoptosis in many cell lines at varying IC_50_ levels (Figure 4e).

In order to further confirm that DUSP26 inhibition is leading to cytotoxicity, we treated IMR32 cells with NSC-87877 and collected cells at time points over a 12-h period. By immunoblot, poly ADP ribose polymerase (PARP) cleavage was seen after 6 h of treatment confirming cell death (Figure 4f, Supplementary Figure 5a). We then tested the phosphorylation status of the two serine DUSP26 target sites on p53 (Ser20 and Ser37).^22^ There was a noticeable increase in Ser37 phosphorylation over the time course. In addition, we saw a significant increase in Ser46 phosphorylation 1 h after treatment compared with the 0 time point (Supplementary Figure 5a). Ser46 is described as an apoptosis activation site for p53 and is a direct target of p38 kinase.^15, 25, 26^ Therefore, in addition to increased Ser46-p53 phosphorylation, we saw an increase in p38 phosphorylation at the activation sites, Thr180/Tyr182, starting at 1 h after treatment with a concomitant increase and stabilization in total p38 (Supplementary Figure 2b). This effect was transient as the level of phosphorylated p38 decreased at 12 h when the most PARP cleavage was seen. This decrease at 12 h was also seen in Ser46-p53, as well (Supplementary Figure 5a). In summary, DUSP26 inhibition with NSC-87877 results in increased phosphorylation of Ser37 and Ser46 on p53, and subsequent cell death as evidenced by increased PARP cleavage.

### Inhibition of p53 or p38 MAPK results in increased cell viability in the setting of NSC-87877 treatment

**Figure 5:**
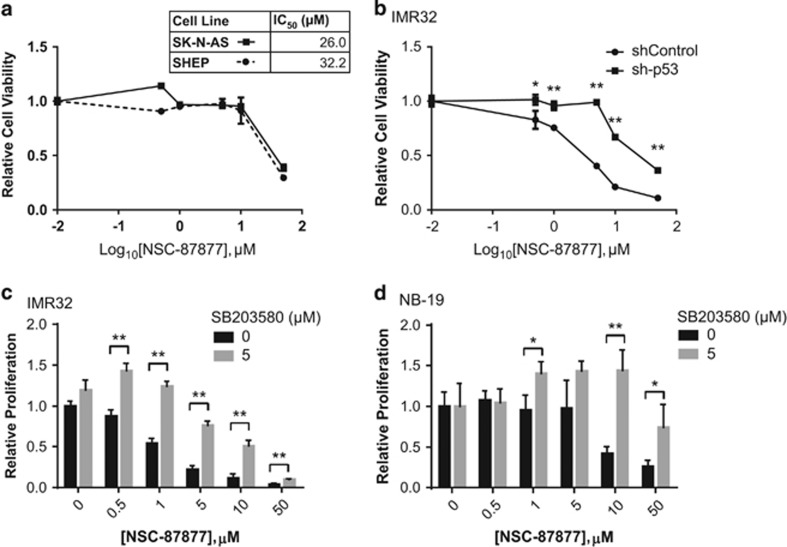
Inhibition of p38 or p53 results in increased cell viability despite treatment with NSC-87877. (**a**) The NB cell lines SK-N-AS and SHEP were seeded into 96-well plates and treated with the indicated concentrations of NSC-87877. After 72 h, the cells were stained with MTT and read at 540 nm absorbance. The data are presented as mean±S.D. The IC_50_ values are show in the corresponding table. (**b**) IMR32 were transduced with a sh-p53 construct, as well as a non-silencing control construct. These transduced cells were seeded into 96-well plates. After 48 h, the cells were stained with MTT and read at 540 nm absorbance. The data are presented as mean±S.D. *P*-values <0.05 (*) or <0.01 (**) are indicated. (**c** and **d**) Two NB cell lines IMR32 and NB-19 were seeded into 96-well plates and treated with 0 or 5μM of the p38 inhibitor SB203580, as well as increasing concentrations of NSC-87877. The data are presented as mean±S.D. *P*-values <0.05 (*) or <0.01 (**) are indicated. All panels are representative for three independent experiments

As NSC-87877 inhibits DUSP26 activity resulting in increased p53 and p38 activation and cell death, we tested if inhibiting either the p53 or p38 pathways in this setting could reverse the cell death. We first sought to determine the effect of NSC-87877 on NB cell lines with defective p53 signaling. The SK-N-AS cell line contains a deactivating deletion at the C-terminus of p53.^27^ The SHEP cell line is another unique cell line with an inactive p53 pathway because of a homozygous deletion of the *CDKN2A* gene resulting in loss of p14^ARF^, a known MDM2 inhibitor.^28^ This results in increased p53 degradation through unregulated MDM2. Both of these cell lines had much higher IC_50_ values (26.03 and 32.24 *μ*M) with NSC-87877 monotherapy than the other cell lines tested (Figures 5a and 4e). From this, we hypothesized that p53 inactivation can also reverse the effects of NSC-87877 treatment. We used an shRNA targeting p53 (sh-p53) to inhibit p53 expression in IMR32 cells and achieved a significant decrease in protein expression (Supplementary Figure 4d). These cells were plated with shRNA non-targeting, control (shRNA control) cells and treated with increasing concentrations of NSC-87877. Figure 5b shows that the sh-p53 cells were much more resistant to cell death than the shRNA control cells with an IC_50_ increasing from 1.37 to 14.7 *μ*M.

In order to test the effects of p38 pathway inhibition on NSC-87877-induced cell death through Ser46-p53 activation, we pre-incubated IMR32 and NB-19 cells with SB203580 (4-[4-(4-fluorophenyl)-2-(4-methylsulfinylphenyl)-1*H*-imidazol-5-yl]pyridine), a well-described p38 inhibitor,^29^ and then exposed them to increasing concentrations of NSC-87877. Figures 5c and d show that the cell viability is significantly increased when the cells are pretreated with the SB203580 inhibitor. The IC_50_ for IMR32 and NB-19 increased from 1.44 to 6.76 *μ*M and 13.1 to >50 *μ*M, respectively, with 5 *μ*M of SB203580.

### DUSP26 regulates p53 and p38 activity in NB

**Figure 6:**
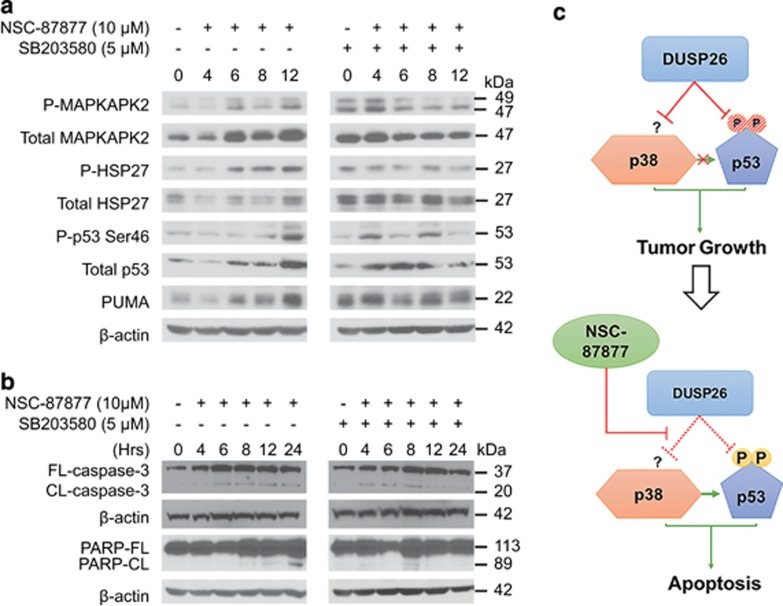
Inhibition of p38 results in decreased expression of p38 and p53 downstream proteins. (**a**) NB-19 cells were treated with NSC-87877 for the indicated times points with and without the presence of the p38 inhibitor SB203580. Immunoblotting was performed using PARP, phospho-MAPKAPK2, MAPKAPK2, phospho-HSP27, HSP27, phospho-p53 serine46, p53 and PUMA antibodies. *β*-Actin was used as a loading control. (**b**) The NB-19 cell line was treated under the same conditions described in panel **a.** Immunoblotting was performed using an antibody specific to caspase-3 (FL-caspase-3) and cleaved caspase-3 (CL-caspase-3). Panels are representative of two independent experiments. (**c**) The inferred mechanism of NSC-87877 action on DUSP26 is outlined. Active DUSP26 causes inactivation of p38 and p53 with their respective downstream proapoptic proteins, resulting in tumor growth. The p53-activating activity of p38 is also suppressed. The addition of NSC-87877 results in competitive inhibition of DUSP26 activity and removes the inhibitory effect of DUSP26 on p38 and p53

Owing to the reversal of cell death with p38 inhibition, we then tested the expression and activation of downstream effector proteins. The NB-19 cell line was treated with 10 *μ*M of NSC-87877 alone or after a pre-incubation period with SB203580. Similar to the previous immunoblot analysis, we again saw in an increase in Ser46-p53 phosphorylation; however, in this cell line we also saw an increase in p53 stability (Figure 6a, Supplementary Figure 5b). With the addition of SB203580, Ser46-p53 phosphorylation decreased as did total p53 levels (Supplementary Figure 5c). In addition, p53 upregulated modulator of apoptosis (PUMA) levels increased with NSC-87877 alone and decreased with SB203580 pre-treatment. With NSC-87877 treatment, the direct downstream target proteins of p38, MAP kinase-activated protein kinase 2 (MAPKAPK2) and heat shock protein 27 (HSP27), had increasing phosphorylation at their respective activation sites, as well as total protein stability. The downstream function of p38 was inhibited by SB203580 pre-treatment as evidenced by decreased phosphorylation of MAPKAPK2 and HSP27. When we analyzed p38 phosphorylation at the known activation sites, Thr180/Tyr182, in these cells, we did not see a robust increase in p38 phosphorylation (Supplementary Figure 2c). As SB203580 only inhibits p38 downstream function and does not affect phosphorylation status of p38,^29^ the p-p38 and total p38 expression looked almost identical in the SB203580/NSC-87877-treated group.

With NSC-87877 treatment and p38 inhibition with SB203580, we tested for expression of cleaved PARP and caspase-3. We again saw an increase in PARP cleavage with increasing time points after administration of NSC-87877, particularly at 8, 12 and 24 h (Figure 6b, Supplementary Figures 5d and e). Confirming that this is indeed apoptosis and not cellular necrosis from small molecule treatment, we found that cleaved caspase-3 (20 kDa) also increased with progressing time points after NSC-87877 treatment (Figure 6b, Supplementary Figures 5d and e). This phenomenon was reversed with the addition of SB203580. These data suggest that DUSP26 most likely has a role in directly inhibiting p53 phosphorylation at Ser37 and indirectly inhibiting p53 phosphorylation at Ser46 by affecting p38 MAPK function in order to promote NB tumor cell growth (Figure 6c).

### NSC-87877 affects NB tumor growth *in vivo*

**Figure 7:**
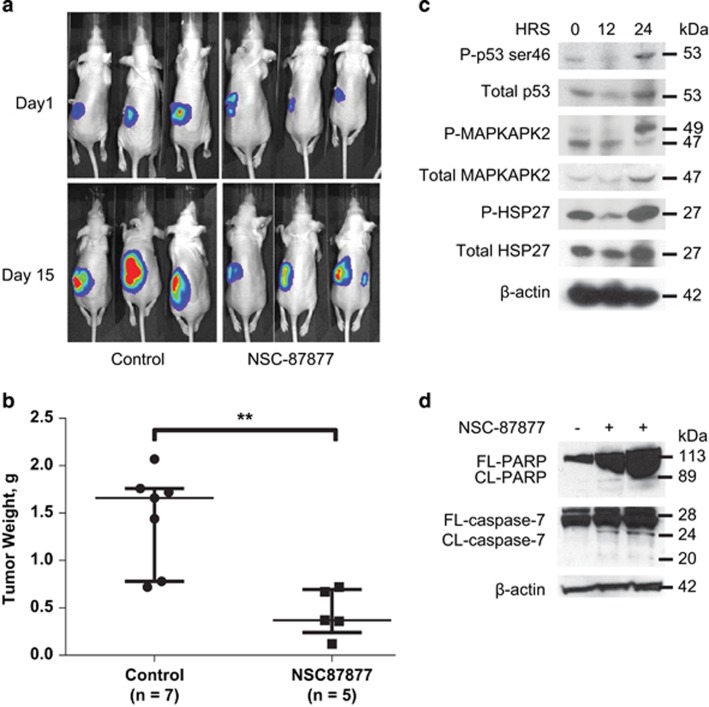
NSC-87877 affects tumor growth, as well as p38 and p53 pathway expression *in vivo*. (**a**) Photos of mice implanted with luciferase-tagged SH-SY5Y visualized after i.p. injection of luciferin. Mice were treated with NSC-87877 (30 mg/kg) via i.p. injection for 15 days and compared with a normal saline vehicle control. (**b**) Graph of tumor weights from control and NSC-87877-treated mice after necropsy. The data are presented as a median±one quartile with individual data points shown. *P*-values <0.01 are indicated (**). (**c**) Three mice were killed at the indicated time points after confirmation of tumor via bioluminescence and treatment with i.p. NSC-87877 (30 mg/kg). Protein was extracted from the tumors. Immunoblotting was performed using phospho-MAPKAPK2, MAPKAPK2, phospho-HSP27, HSP27, phospho-p53 serine 46 and total-p53 antibodies. *β*-Actin was used as a loading control. (**d**) One tumor sample from a mouse treated with carrier control, and two tumor samples from mice treated with NSC-87877 (30 mg/kg) via i.p. injection for 15 days were harvested. Proteins were extracted from the tumors and immunoblotting was performed using antibodies specific to PARP and caspase-7. *β*-Actin was used as a loading control. Panels **a-c** are representative of two independent experiments. Panel **d** is representative of two independent experiments

In order to test the effect of small molecule inhibition of DUSP26 *in vivo*, we used a well-established intrarenal NB tumor mouse model.^30^ SH-SY5Y cells with luciferase expression levels were injected into the left kidney of female nude mice. After 10 days of tumor growth, mice were treated with i.p. injection of placebo control (control) or 30 mg/kg of NSC-87877 once daily for 15 days. Mice were monitored weekly with i.p. injection of luciferin and bioluminescence imaging. Figure 7a demonstrates equivalent tumor burden with similar bioluminescent signal at day 1 of therapy and a difference in signal at 15 days of therapy. On day 15, necropsy was performed and the tumors were weighed. The control group tumors were significantly larger than the NSC-87877-treated group (*P*<0.01; Figure 7b).

In order to test phosphorylation status of p38 and p53 *with in vivo* NSC-87877 treatment, we generated xenografts with SH-SY5Y luciferase-tagged cells as above and grew the tumors for 14 days. After confirming a significant tumor size by bioluminescence, three mice were treated with an i.p. injection of NSC-87877 (30 mg/kg). The mice were placed under anesthesia and a piece of tumor was harvested through the same flank incision as the intrarenal implantation at time points. By immunoblot, the tumors displayed increased phosphorylation and stabilization of Ser46-p53, total p53, p-MAPKAPK2, total MAPKAPK2, p-HSP27 and total HSP27 as seen in the *in vitro* experiments (Figure 7c, Supplementary Figure 5f). We also found increased p38 phosphorylation (Thr180/Tyr182) at 24 h of treatment with NSC-87877 (Supplementary Figure 2d). Tumor samples harvested after 15 days of NSC-87877 treatment from the experiment in Figure 7a were tested for PARP and caspase-7 cleavage to show presence of cellular apoptosis *in vivo*. In two of the experimental samples, we saw more PARP and caspase-7 cleavage when compared with the control sample (Figure 7d). In summary, we demonstrated a significant decrease in tumor growth in NSC-87877-treated mice and showed a similar mechanism of p53 and p38 activation resulting in cellular apoptosis.

## Discussion

NB continues to be a prevalent cancer in children with a poor prognosis in 50% of patients because of treatment refractory and/or metastatic disease.^1^ Although intensive chemotherapy and immunotherapy have modestly improved survival, the associated increase in toxicity of our current approach suggests that future improvements in cure rates will require novel molecular targeted approaches. One approach to targeted therapy is to activate intrinsic death pathways, such as the p53 tumor-suppressor pathway. Several studies have investigated the mechanisms repressing p53 activation in NB. Key inhibitory proteins such as PARC, MDM2 and apoptosis-antagonistic transcription factor (AATF) have been described to sequester, degrade or suppress transcription factor activity of the p53 protein.^31, 32, 33, 34^

The results of our study suggest that DUSP26 is an important regulator of p53-mediated apoptosis in NB. The proposed mechanism of action is similar to how Wip-1 phosphatase (*PPM1D*) is described as a deactivator of p38 and in turn p53 by inhibiting Ser46-p53 phosphorylation in breast cancer.^15^ In our previous studies, we have established that DUSP26 expression is highly specific to NB and is preferentially overexpressed in high-risk NB patients with the worst outcome.^18, 22^ The identified substrates for DUSP26 are p38 and p53 both of which are strong tumor suppressors in NB and many other cancers.^35^ In this study, we were able to show that the DUSP26 target site Ser37-p53 shows increased phosphorylation indicating p53 activation with NSC-87877 treatment. Moreover, the Ser46 site also undergoes increased phosphorylation suggesting that p38 MAPK is also activated with NSC-87877 treatment.^36^ This was supported by downstream activation of MAPKAPK2 and HSP27. In addition, p38 and/or p53 inhibition resulted in a significant reversal of the cell death seen with NSC-87877 treatment strongly suggesting that DUSP26 inhibition results in activation of these tumor-suppressor pathways.

This article focuses on the use of a small molecule inhibitor, NSC-87877, which targets and inhibits DUSP26 function.^20^ Our data show that NSC-87877 causes a proliferation defect in NB cell lines similar to knocking down expression with DUSP26-specific shRNA. In regards to ‘off target effects' of using NSC-87877, silencing SHP-1 expression resulted in a pro-proliferative phenotype in contrast to that seen with DUSP26 knockdown suggesting that NSC-87877 specifically targets DUSP26 in NB with less affect on other known substrates. Even with the validation data presented, it is still quite possible that NSC-87877 activates other pathways than what we have described. However, our experiments showing that p53 inhibition with shRNA and p38 MAPK inhibition with SB203580 both partially reverse the cytotoxicity seen with NSC-87877 treatment, strongly suggest that NSC-87877 inhibits DUSP26 function resulting in unregulated p53 activation and cell death.

In our experiments analyzing the regulation of the p53 and p38 pathways by DUSP26 inhibition with NSC-87877, we were not able to show a consistent pattern of p38 phosphorylation. Several experiments both *in vitro* and *in vivo* showed that p38 phosphorylation increased with NSC-87877 treatment (Supplementary Figures 2a, b, and d) similar to previously published reports.^25^ However, significant p38 phosphorylation changes were not seen in the NB-19 cell line treated with NSC-87877 (Supplementary Figure 2c). Instead, significant changes in Ser46-p53, total p53, PUMA, MAPKAPK2 and HSP27 were seen. These data suggest that DUSP26 may have a unique regulation of p38 that is not yet undescribed. The experiments with the IMR32 cell line also revealed increasing total p38 stability accompanying increasing phosphorylation of p38 at Thr180/Tyr182 with NSC-87877 treatment. This finding is unique and we feel possible because phosphorylation of many enzymatic proteins leads to stability. Other studies have shown discrepancies in total p38 stability with activating phosphorylation thus demonstrating that the mechanisms behind this regulation are not known and can vary with cell types.^37, 38^

Some controversy has been raised in the literature on the mechanism of action of DUSP26 on the MAPK's. Patterson *et al.*^39^ state in their paper that DUSP26 does not function in a MAPK-dependent manner and does not dephosphorylate p38. They also state that DUSP26 is not overexpressed in NB cell lines by qPCR. In addition to our studies showing that DUSP26 clearly dephosphorylates p38, multiple other studies have shown p38 dephosphorylation with DUSP26 overexpression.^18, 20, 21^ As far as expression in NB, our group has clearly shown by immunohistochemistry that DUSP26 is overexpressed in NB tissue samples *versus* normal adrenal gland controls particularly in high-risk patients. Studies in the literature also describe DUSP26 as a potential tumor suppressor in breast cancer and cervical carcinoma.^39, 40^ This data, however, convincing and well described, does not mandate that DUSP26 must serve as a tumor suppressor in every cancer especially when comparing an embryonal, neural crest-derived malignancy to epithelial malignancies seen in adults.

Currently, targeted molecular therapies for NB are limited. The active trials through the Children's Oncology Group utilize anti-GD2 inhibition with monoclonal antibody, ALK inhibition with crizotunib and differentiation induction with retinoic acid.^1, 2^ Agents targeting the p53 pathway show promise, such as, RG7112 and RITA targeting the MDM2-p53 complex, P22077 targeting USP7 (a p53 regulating ubiquitin-specific protease), miRNA antagonist targeting miR-380-5p (an epigenetic regulator of p53) and NSC697923 targeting UBE2N (a p53 structural modulator).^6, 33, 41, 42, 43^ The data presented in this article suggest that DUSP26 inhibition is a potential therapeutic strategy for NB targeting both the p53 and p38 tumor-suppressor pathways. Our hope is that this study will spawn an interest in DUSP26 inhibition to help develop novel therapies for this devastating disease.

## Materials and Methods

### Antibodies and reagents

NSC-87877 (565851) was obtained from Calbiochem (EMD Millipore, Billerica, MA, USA). The p38 inhibitor, SB203580 (sc-3533) was obtained from Santa Cruz Biotechnology (Dallas, TX, USA). Anti-DUSP26 was obtained from Novus Biologicals (NBP1-31254) (Novus Biologicals, Littleton, CO, USA) and Abcam (ab22141) (Abcam Inc., Cambridge, MA, USA). Monoclonal anti-*β*-actin (A2228) was obtained from Sigma-Aldrich (St. Louis, MO, USA). Anti-rabbit IgG (7074S), anti-mouse IgG (7076S), anti-phospho-p53-ser20 (9287), anti-phospho-p53-ser37 (9289), anti-phospho-p53-ser46 (2521), anti-MAPKAPK-2 (3042), anti-phospho-MAPKAPK-2 Thr334 (3041), anti-PARP (9542), anti-PUMA (D30C10), anti-phospho-HSP27 Ser82 (2401), anti-HSP27 (2402), anti-caspase-7 (9492) and anti-caspase-3 (9662S) antibodies were obtained from Cell Signaling Technology (Danvers, MA, USA).

### Cell lines

NB cell lines that were MYCN amplified (IMR32, LAN-1, NB-19, SMS-KCN, JF, NGP) and non-MYCN amplified (SH-SY5Y, SH-SY5Y-Luc, SK-N-AS, SHEP, SK-N-SH, CHLA-225, NB16) were used in this study. The SH-SY5Y cell line was cultured in 50% MEM and 50% Ham's F12. IMR32 and SK-N-SH were grown in MEM. RPMI 1640 medium was used to culture SH-SY5Y-Luc, NGP, JF, SHEP, NB16 and NB-19. DMEM medium was used to culture SK-N-AS, LAN-1 and SMS-KCN. CHLA-225 was grown in IMDM medium. All media were supplemented with 10% heat-inactivated, fetal bovine serum (FBS, SAFC Biosciences, Lenexa, KS, USA), 100 units/ml streptomycin/penicillin and 2 mM glutamine (Thermo Fisher Scientific, Waltham, MA, USA). All cells were incubated at 37 °C in a humidified atmosphere of 5% CO_2._

### Proliferation and cell viability assays

To test proliferation, the cell lines IMR32, SH-SY5Y and NB-19 were plated in 96-well plates with 1 × 10^3^ cells per well. After cells were allowed to settle for 24 h, NSC-87877 (0, 0.025 and 0.05 *μ*M) was added. An MTT assay was performed for consecutive days by replacing the media in each well with 9% MTT (5 mg/ml)/media (v/v). After 4-h incubation at 37 °C, 85 *μ*l of MTT/media was aspirated and 50 *μ*l of DMSO was added. The plate was then read at 550 nm in a multimode plate reader (Beckman Coulter, Brea, CA, USA) within 10 min. In experiments using shRNA knockdowns in place of a chemical inhibitor, cell proliferation was measured using Cell Counting Kit-8 (CCK-8) (Dojindo Molecular Technologies Inc., Rockville, MD, USA) after seeding 96-well plates with 1 × 10^3^ cells per well as per the manufacturer's instructions in place of MTT.

### Colony formation assay

For the soft agar assay, a base layer of 1% (w/v) agarose/cell culture media was plated into six-well plates and allowed to solidify. A top layer of NB cells were resuspended in a mixture of 0.7% (w/v) agarose/cell culture media to give a final concentration of 20 000 cells/2 ml. After 2–3 weeks, 1 ml of 5 mg/ml MTT was added to each well and incubated at 37 °C for 1 h. Photos were then taken of each well. The NIST Integrated Colony Enumerator (National Institute of Standards and Technology, Gaithersburg, MD, USA) was used to count the colonies in each plate.

### RNA interference, overexpression constructs and retroviral/lentiviral transduction

The shRNA target sequences used were as follows: pSuper-Scrambled control (sh-Control), 5′-CGTCTTTTCGGACTTAGAGAG-3′ pSuper-DUSP26-1 (sh-DUSP26-1), 5′-AAGACAGCCTGTAACCATGCC-3′. These constructs were cloned into the indicated shRNA vector. Each knockdown vector was paired with its respective scrambled control. The pLSLPw-Scrambled (sh-Control) and pLSLPw-p53 (sh-p53) used in the p53 knockdown experiments were cloned and generously contributed by AV Budanov, as described.^44^ Viral supernatant was made by co-transfecting HEK-293t cells with the packaging vectors RDF and Pegpam3 using polyethylenimine (PEI) (1 *μ*g/*μ*l) added at a 3 : 1 ratio of PEI (*μ*g):total DNA (*μ*g). These shRNA viral particles were transduced into IMR32 and/or SH-SY5Y with 8 *μ*g/ml of hexadimethrine bromide (Polybrene, H9268, Sigma-Aldrich), and stable cell lines were established after 1 week of puromycin (2 *μ*g/ml) selection. Knockdown was confirmed using real-time PCR (qPCR) or immunoblotting. With these cell lines, we studied the effects of DUSP26 knockdown on anchorage-independent growth, cell cycle and *in vivo* tumor growth using an orthotopic NB mouse model.

### Cytotoxicity assays using NSC-87877 on NB cell lines

The cell lines IMR32, SH-SY5Y, NB-19, LAN-1, SK-N-AS, SK-N-SH and SHEP were individually plated at 1 x 10^4^ cells per well in flat bottom 96-well plates to evaluate the cytotoxicity of NSC-87877, with and without doxorubicin. Treatment was initiated after cells were allowed to settle for 24 h. Plates were then read between 24 and 48 h after the two drugs were added. An MTT assay was performed using a multimode plate reader (Beckman Coulter) as described in the proliferation and cell viability assay. This experiment was repeated using IMR32 and NB-19 using NSC-87877 and the p38 inhibitor SB203580 (0 and 5 *μ*M).

### Quantitative reverse transcription-PCR

Total RNA was extracted from cell lines using Direct-zol RNA Miniprep Kit as per the manufacturer's instructions (cat# 11–331, Zymo Research, Irvine, CA, USA), and RNA purity and quantity were determined using a spectrophotometer measuring absorbance at 260/280 nm. SYBR green primers were designed to measure the quantity of specific mRNA, which are as follows: DUSP26 mRNA (forward, 5′-GCAGCGCTTGCAAAGAGCAG-3′ reverse, 5′-ACAGGAGACCTTGAGCTACT-3′). Glyceraldehyde 3-phosphate dehydrogenase was used as the internal control.

### Immunoblotting assay

For immunoblotting, IMR32 and NB-19 cells were cultured for 48 h. All agents were added while cells were in logarithmic growth phase. For IMR32, NSC-87877 (50 *μ*M) was added to each plate and cells were collected at 0, 1, 2, 4, 6, 8 and 12 h. For NB-19, NSC-87877 (10 *μ*M) and a vehicle control was added to respective plates, and then collected at various time points (0–12 h). In a subsequent experiment, the p38 inhibitor, SB203580, and a vehicle control was added to each plate. After 12 h of treatment, NSC-87877 was added to all plates that were collected at various time points (0–12 h). The cell proteins were lysed with protein lysis buffer (50 mM Tris-HCl at pH 7.4, 150 mM NaCl, 1 mM EDTA, 1% NP-40, 0.25% sodium deoxycholate, 1 mM phenylmethylsulfonyl fluoride, 1 mM benzamidine, 10 mg/ml leupeptin, 1 mM dithiothreitol, 50 mM sodium fluoride, 0.1 mM sodium orthovanadate and phosphatase inhibitor cocktail 2 and 3 (p5726 and p0044, Sigma-Aldrich)) for 20 min at 4 °C. Equal concentrations of protein extracts (100 *μ*g) were resolved by sodium dodecyl sulfate-PAGE and transferred to a nitrocellulose membrane (Bio-Rad, Hercules, CA, USA). Depending on the antibody, the membrane was blocked with buffer containing 5% non-fat milk in TBST (10 mM Tris, pH 7.8, 150 mM NaCl and 0.05% Tween) or 5% bovine serum albumin (A9418) (Sigma-Aldrich) in TBST for 1 h at room temperature. The specific antibody was then incubated with the membrane at 4 °C overnight. This was then followed by incubation with horse radish peroxidase (HRP)-conjugated, anti-mouse secondary antibody (Ab) or HRP-conjugated, anti-rabbit secondary antibody (Ab). The membrane was developed using the Pierce ECL Western Blotting Substrate chemiluminescent detection system (#32106) (Thermo Fisher Scientific).

### Effect of RNA interference and NSC-87877 on NB cell growth in an orthotopic mouse model

Female Nu-nude mice (Taconic Biosciences, Hudson, NY, USA) were used for *in vivo* testing of NSC-87877 compared with control. SH-SY5Y transduced luciferase cells (SY5Y-Luc) and SH-SY5Y transduced with shDUSP26-1 were implanted into the left kidney as previously described.^30^ The mice were imaged 10 days after implantation and flux measured. A threshold of 5 × 10^7^ total flux (p/s) was used to standardize the mice who would be treated. Two groups were treated, one with NSC-87877 using a dose of 30 mg/kg/day, and the other with a carrier control composed of an equivalent volume of 0.9% NaCl via i.p. injection. After 15 days, necropsy was performed and tumor weights measured. For phosphor-immunoblotting, SH-SY5Y-Luc were implanted into the kidneys of three mice, once the previously indicated threshold for flux was reached, two mice were treated with 30 mg/kg of NSC-87877 and one control mouse was treated with carrier control. The mice were killed, at 12 and 24 h after administration of NSC-87877, and necropsy was performed. Tumors were immediately flash frozen with liquid nitrogen for later protein extraction. Protein was extracted by grinding 10 mg of tumor tissue, which was mixed with protein lysis buffer, passed through a 22 G needle, and incubated on ice for 30 min.

### Statistical analysis

A two-tailed Student's *t*-test was used to determine statistical significance of *in vitro* cell viability and cytotoxicity experiments. The Kruskall–Wallis test was used to determine the statistical significance of *in vivo* tumor size differences between control and treatment groups. IC_50_ were calculated using nonlinear regression in Prism (Graphpad, La Jolla, CA, USA). A *P*-value <0.05 was considered statistically significant.

## Abbreviations

NB: neuroblastoma
DUSP: dual specificity protein phosphatase
PARP: poly ADP ribose polymerase
NSC-87877: 8-Hydroxy-7-[(6-sulfo-2-naphthyl)azo]-5-quinolinesulfonic acid
SB203580: 4-[4-(4-fluorophenyl)-2-(4-methylsulfinylphenyl)-1*H*-imidazol-5-yl]pyridine
MAPK: mitogen-activated protein kinase
MDM2: mouse double minute 2
SHP: src homology region 2 domain-containing phosphatase
VP-16: etoposide
MTT: (3-(4,5-Dimethylthiazol-2-yl)-2,5-Diphenyltetrazolium Bromide)
MAPKAPK: MAP kinase activated protein kinase
HSP27: heat shock protein 27
PUMA: p53 upregulated modulator of apoptosis
i.p.: intraperitoneal
