# Supplementary material for: NSC-87877 inhibits DUSP26 function in neuroblastoma resulting in p53-mediated apoptosis
Source: Cell Death Dis. 2015 Aug 6;6(8):e1841–. doi: 10.1038/cddis.2015.207 (PMC4558500; doi:10.1038/cddis.2015.207)
Supplement: Supplementary Information [file cddis2015207x1.doc]

**Supplemental Materials and Methods:**

**Antibodies and reagents.** Anti-p38 MAPK (9212) and anti-phospho-p38 MAP Kinase Thr180/Tyr182 #9211) were obtained from Cell Signaling Technology (Cell Signaling Technology, Danvers, MA). Anti-N-Myc (OP13) was obtained from EMD Millipore (EMD Millipore, Darmstadt, Germany). Anti-p53 (DO-1, sc-126) and anti-SHP1 (C-19, sc-287) antibodies were obtained from Santa Cruz Biotechnology (Santa Cruz Biotechnology, Dallas, TX).

**Cell culture, immunoblotting, proliferation, colony formation, and real time PCR.** These assays were performed as described in the methods section of the manuscript.

**Densitometry.** Densitometry was performed using ImageJ (National Institutes of Health, [http://imagej.nih.gov](http://imagej.nih.gov/)).

**Crystal violet clonogenic assay.** For the clonogenic assay, 3 x 10^4^ cells/well were plated into six well plates using the appropriate media. After incubation for two weeks the colonies were washed with room temperature PBS. The colonies were incubated in 0.05% crystal violet (C0775, Sigma-Aldrich, St Louis, MO), for 10 minutes, washed with distilled water, allowed to air dry overnight, then photographed.

**RNA interference, overexpression constructs, and retroviral/lentiviral transduction.** The shRNA target sequences used were as follows: pSuper-Scrambled control (sh-Control), 5’-CGTCTTTTCGGACTTAGAGAG-3’; pSuper-SHP-1 (sh-SHP1), 5’-GCAGGAGGTGAAGAACTTGT-3’; TRC2-pLKO-puro-Scrambled (sh-Control), 5’- CGTCTTTTCGGACTTAGAGAG -3’; TRC2-pLKO-puro-DUSP26-2 (sh-DUSP26-2), 5’- AACATACAGAGGTGCATGGCT-3’; TRC2-pLKO-puro-DUSP26-3 (sh-DUSP26-3), 5’-ACAGGAGACCTTGAGCTACT. Stable clones and confirmation of knockdown were performed as outlined in the manuscript.

**Supplemental Figure Legends:**

**Supplemental Figure 1** DUSP26 is expressed in neuroblastoma cell lines. (**a**) Protein was extracted from a panel of 12 NB cell lines. Immunoblotting was performed using DUSP26, SHP1, and N-Myc antibodies. β-actin was used as a loading control. (**b,c**) Densitometry showing protein expression relative to β-actin was performed on the immunoblotting results shown in panel **a**.

**Supplemental Figure 2** NSC-87877 modulates DUSP26 mediated p38 phosphorylation. Immunoblotting was performed with p38 and phospho-p38 antibodies on (**a**) SK-N-AS cells transduced with a DUSP26 expression construct and empty control plasmid treated with 10 µM of NSC-87877 for 12 h; (**b**) IMR32 cells treated with 50 µM NSC-87877 for the indicated durations; (**c**) NB-19 cells treated with NSC-87877 for the indicated times points with and without the presence of the p38 inhibitor SB203580. β-actin was used as a loading control. (**d**) Three mice implanted with luciferase tagged SH-SY5Y were sacrificed at the indicated time points after confirmation of tumor via bioluminescence and treatment with i.p. NSC-87877 (30 mg/kg). Immunoblotting was performed using p38 and phospho-p38 antibodies. β-actin was used as a loading control.

**Supplemental Figure 3** Knockdown of DUSP26 causes decreased cell proliferation in NB cell lines. (**a**) IMR32 was transduced using an shRNA construct targeting DUSP26 as well as a non-silencing construct. Real-time PCR was used to quantify DUSP26 mRNA. Results were presented as relative fold change compared to shC ± SD. *P*-values <0.01 (**) are indicated. (**b**) IMR32 transduced with shC and shD26-1 were each seeded into individual 96-well plates. Cell proliferation was measured by CCK-8 at 24 h, and at every subsequent 24 h. Results are presented as mean ± SD. *P*-values <0.05 (*) are indicated. (**c**) IMR32 and SH-SY5Y were transduced using two unique shRNA constructs targeting DUSP26 (shD26-2 and shD26-3) as well as a non-silencing construct. Real-time PCR was use to quantify DUSP26 mRNA. Results were presented as relative fold change compared to shC ± SD. *P-*values <0.01 (**) are indicated. (**d**) The knockdown cells referred to in panel **c** were individually plated into six well plates and incubated in the appropriate media for two weeks. The colonies were then fixed and stained with crystal violet.

**Supplemental Figure 4** Knockdown of SHP1 does not cause a proliferation defect in NB. (**a**) Protein was extracted from SH-SY5Y transduced with a SHP1 shRNA construct. Immunoblotting was performed using a SHP1 antibody. β-actin was used as a loading control. (**b**) IMR32 transduced with shC, shD26-1, and shSHP-1 were each seeded into individual 96-well plates. Cell proliferation was measured by CCK-8 starting at 24 h, and at every subsequent 24 h. Results are presented as mean ± SD. *P*-values <0.05 (*) and <0.01 (**) are indicated. (**c**) SH-SY5Y transduced with shSHP1 was seeded into 6-well plates with media and agar, and then grown for 2 weeks. The colonies were stained with MTT for 4 h. (**d**) IMR32 was transduced using an shRNA construct targeting p53 as well as a non-silencing construct. Cell pellets were collected and immunoblotted using an antibody specific to p53.

**Supplemental Figure 5** Quantification of p53 and p38 downstream targets after treatment of NB cell lines with NSC-87877. (**a**) Densitometry was performed on the immunoblotting results shown in Figure 4f for IMR32 cells treated with NSC-87877. (**b**) Densitometry results for Figure 6a showing NB-19 cells treated with NSC-87877 alone and (**c**) in combination with SB203580. (**d**) Densitometry for cleaved PARP and cleaved caspase-3 for NB-19 cells treated with NSC-87877 alone and (**e**) in combination with SB203580. (**f**) Densitometry showing protein expression on results shown in Figure 7c. All densitometry values are shown relative to β-actin.
