# Supplementary figures and images for: NSC-87877 inhibits DUSP26 function in neuroblastoma resulting in p53-mediated apoptosis
Source: Cell Death Dis. 2015 Aug 6;6(8):e1841–. doi: 10.1038/cddis.2015.207 (PMC4558500; doi:10.1038/cddis.2015.207)

a)

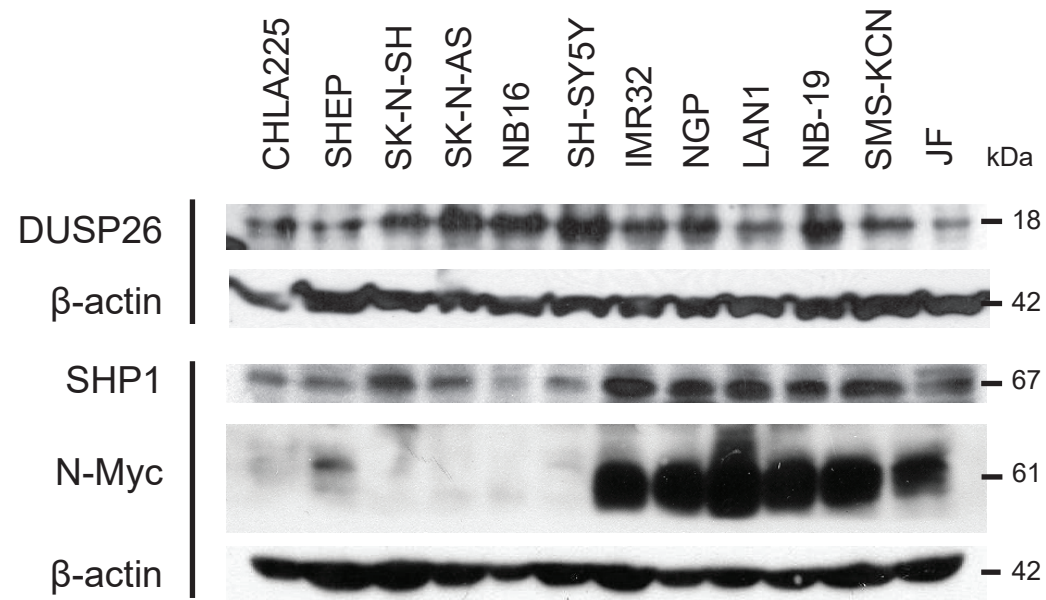

b)

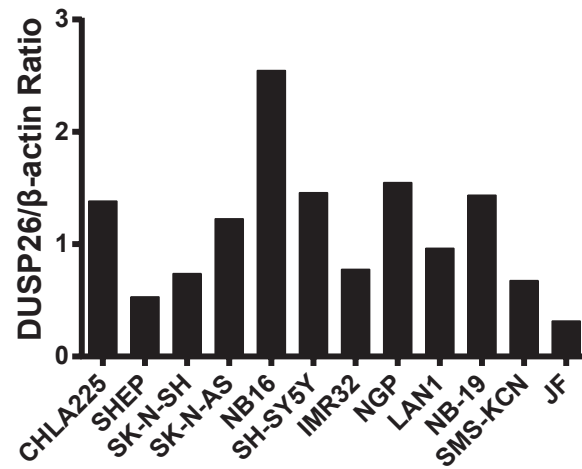

c)

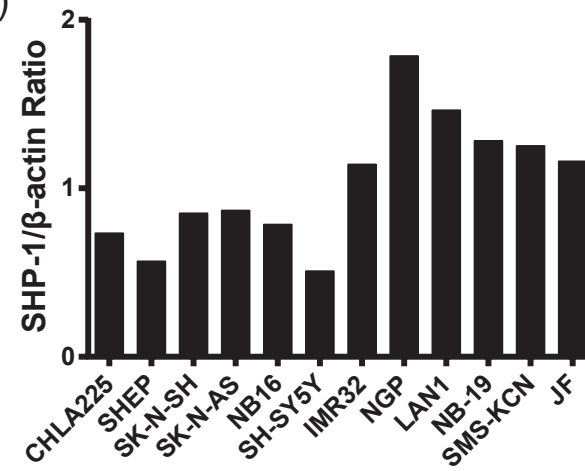

Supplement: Supplementary Figure 1 [file cddis2015207x2.pdf]

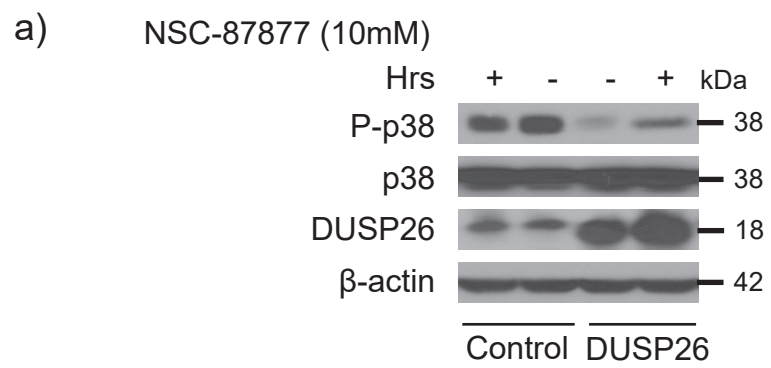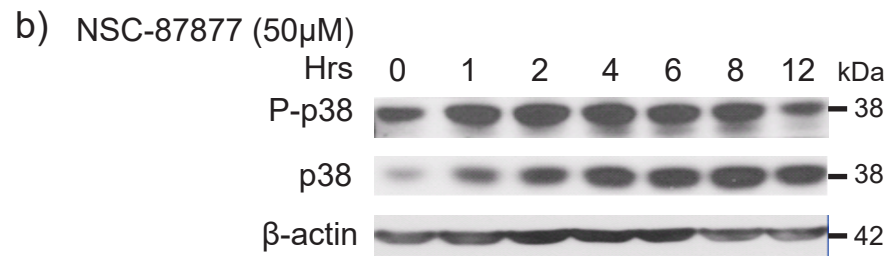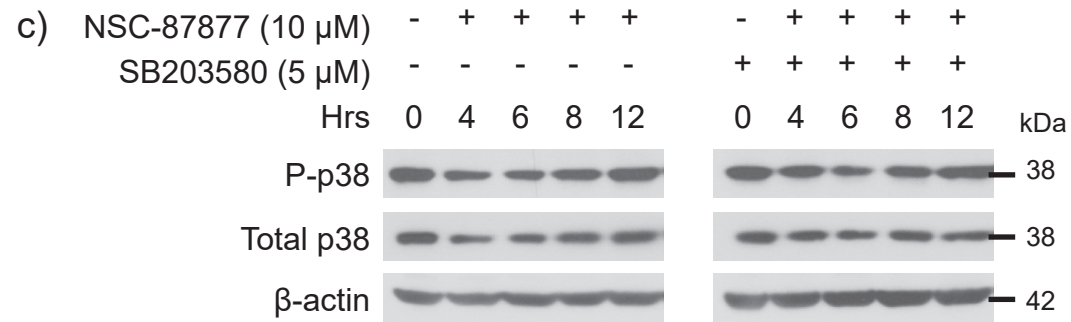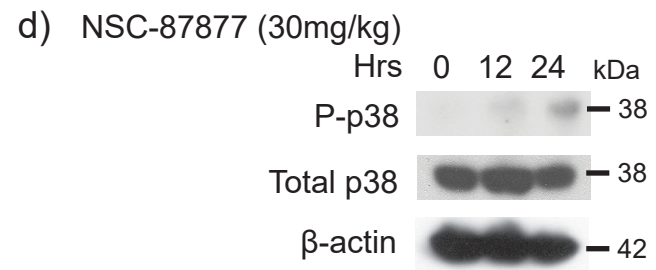

Supplement: Supplementary Figure 2 [file cddis2015207x3.pdf]

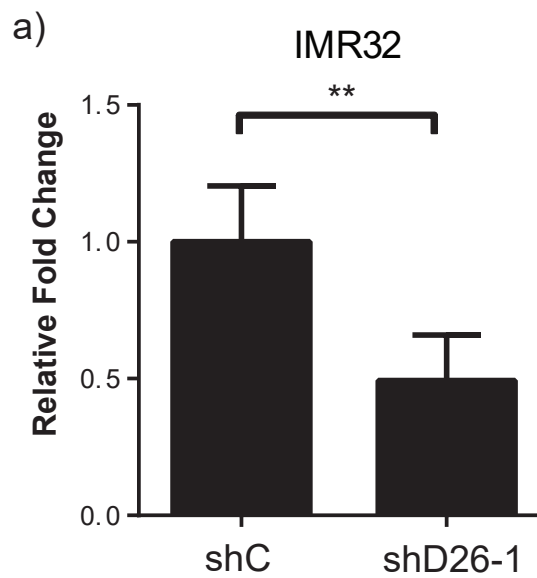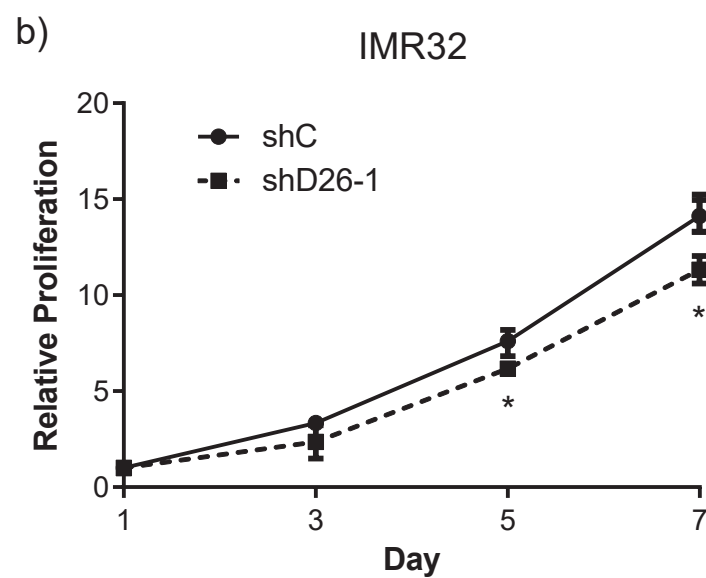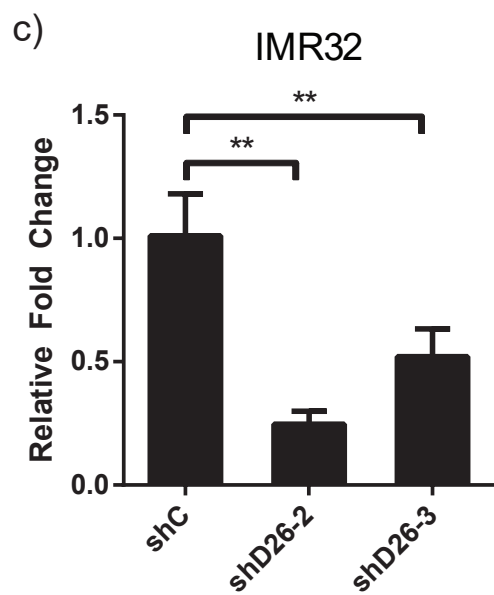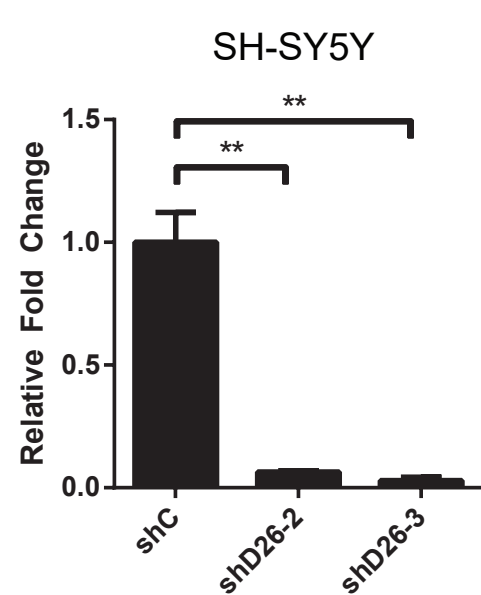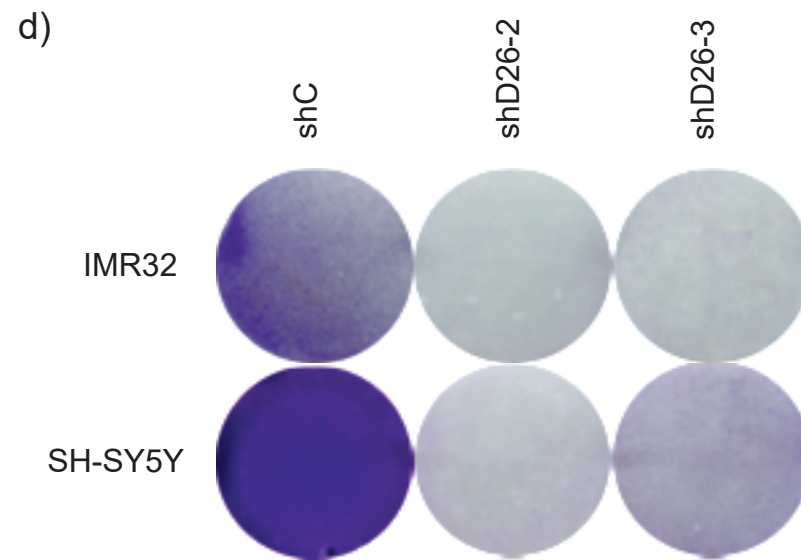

Supplement: Supplementary Figure 3 [file cddis2015207x4.pdf]

a)

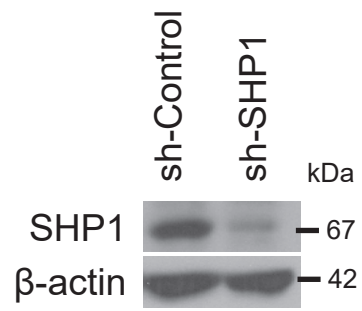

b)

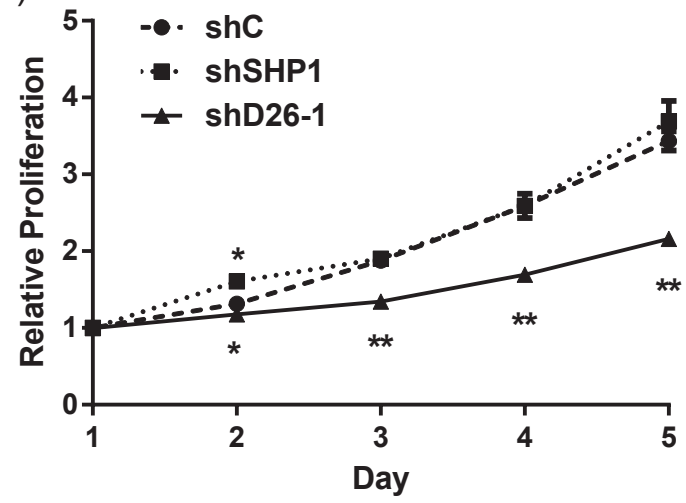

c)

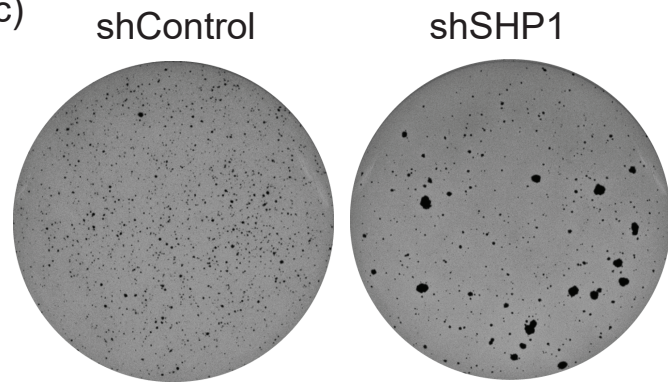

d)

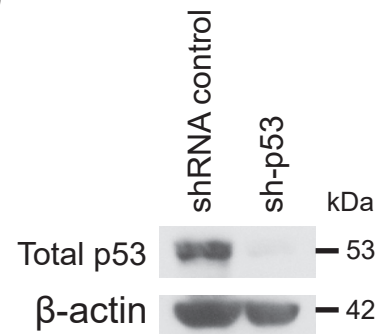

Supplement: Supplementary Figure 4 [file cddis2015207x5.pdf]

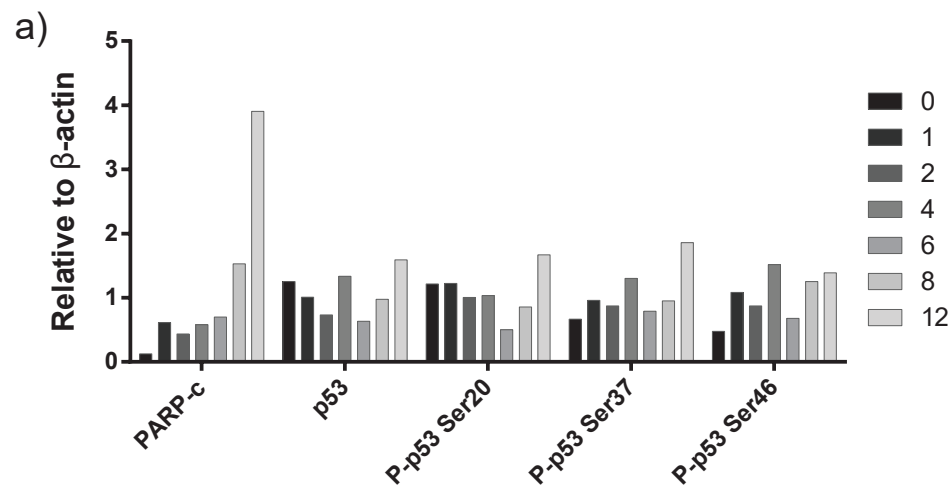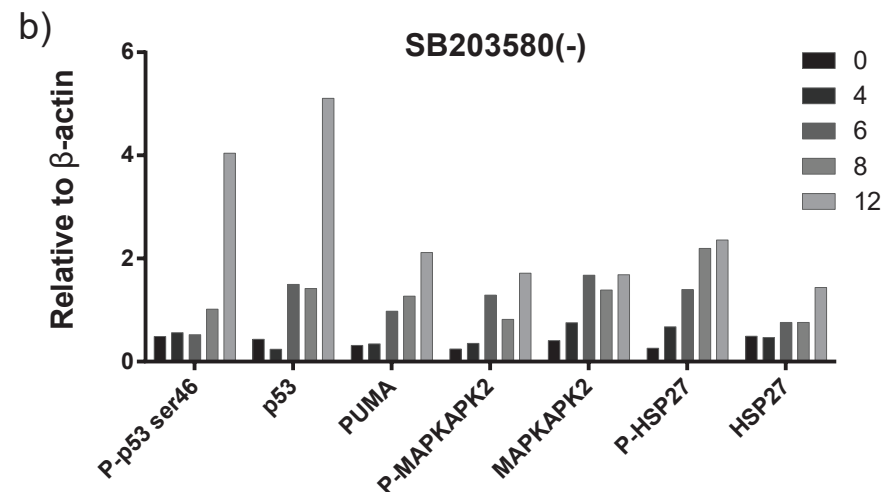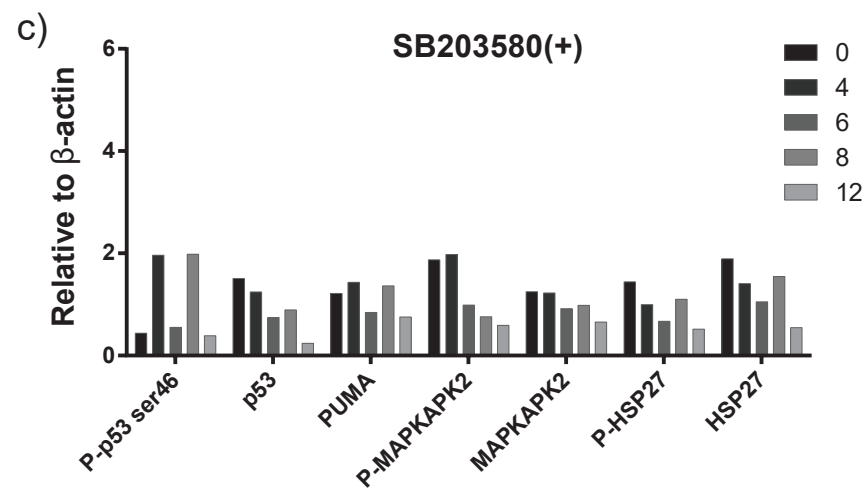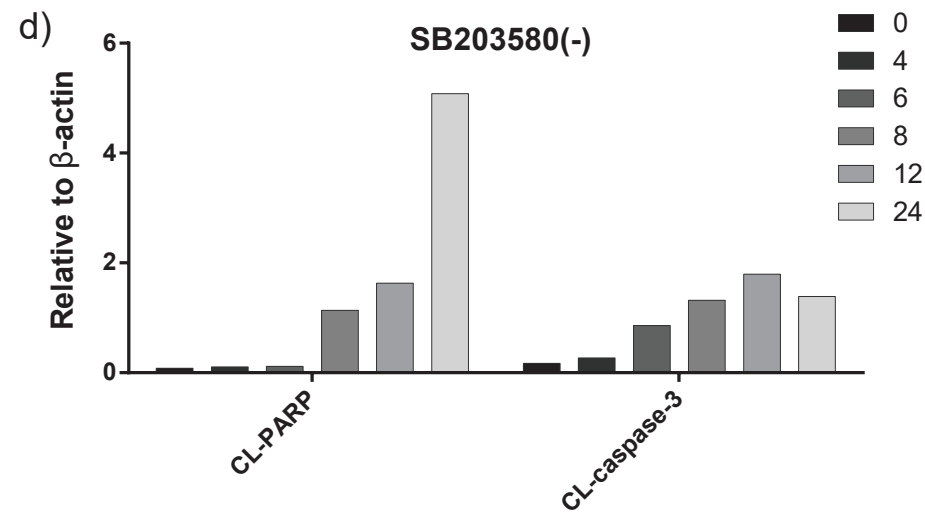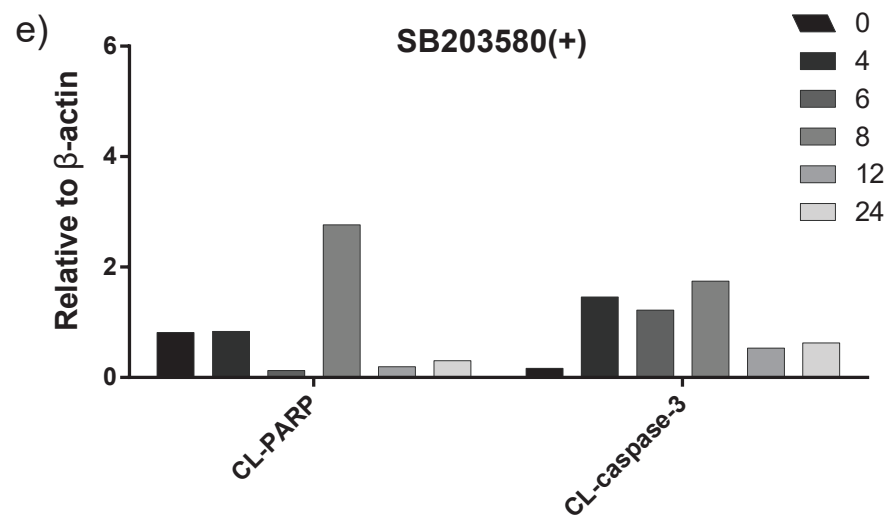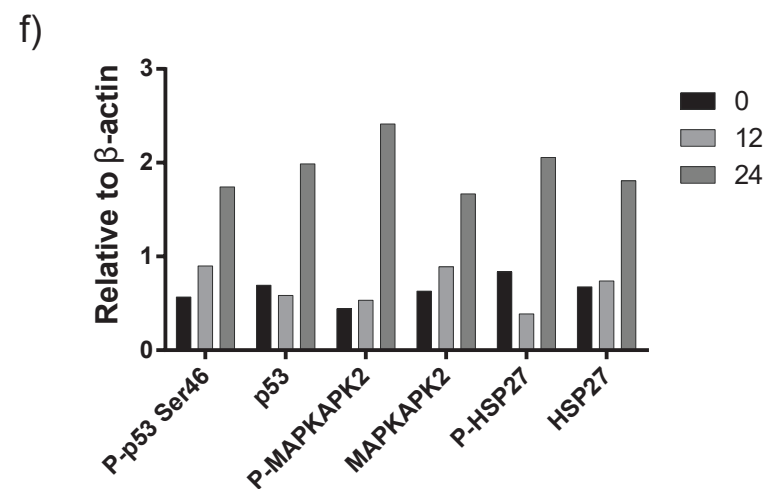

Supplement: Supplementary Figure 5 [file cddis2015207x6.pdf]
